# Eccrine Porocarcinoma with Zosteriform Metastasis

**DOI:** 10.7759/cureus.6873

**Published:** 2020-02-04

**Authors:** Carlos Lehmann, Paola Rodriguez Ossa, Maria Vargas Manrique, Alvaro E Acosta, Yesid Quintero Pérez

**Affiliations:** 1 Breast and Soft Tissue Surgery, National Cancer Institute, Bogotá D.C., COL; 2 Pathology, National Cancer Institute, Bogotá D.C., COL; 3 Dermatology, The National University of Colombia, Bogotá D.C., COL; 4 Surgery, South Colombian University, Neiva, COL

**Keywords:** porocarcinoma, metastasis, zosteriform

## Abstract

Eccrine porocarcinoma is a rare malignant tumor that develops in the eccrine glands, appearing as a primary tumor, or by malignant transformation of an eccrine poroma. It is a carcinoma with high metastatic and recurrent potential; it has the same incidence in both sexes, and mainly affects the elderly. Its diagnosis, rather than clinical, is histological, and due to the rarity of the disease, it is a pathological challenge. There are no standardized treatment guidelines for porocarcinoma, but surgical resection with tumor-free margins is considered the basis of treatment, in addition to sentinel node biopsy under risk factors and individualization of each patient. For the metastatic form, chemotherapy and radiotherapy are the treatment of choice. Herein, we present the case of a man with eccrine porocarcinoma with extensive zosteriform skin metastasis and lymph node involvement, treated with chemotherapy and concomitant radiotherapy.

## Introduction

Eccrine porocarcinoma is a rare skin carcinoma, also known as malignant hidroacanthoma simplex, sweat gland carcinoma, malignant syringoacanthoma, or dysplastic poroma. It occurs in the intraepidermal portion of the sweat gland duct. Its behavior is locally aggressive, with high recurrence rates (20%), and metastases to lymph nodes (20%) and to distant solid organs (10%). This tumor may occur de novo or develop from a preexisting lesion. Its slow asymptomatic growth, in addition to the low incidence of the disease, conditions a late clinical and histological diagnosis [1]. The prognosis of porocarcinoma is difficult to establish due to the lack of follow-up of the cases described in the literature and the low incidence of the tumor.

Nodal involvement is the main and most frequent form of metastatic presentation of the porocarcinoma, with cutaneous metastasis being an unusual type, and zosteriform presentation being even more so with little literature reported [1]. Therefore, we present a case of zosteriform type metastatic eccrine porocarcinoma of the skin and lymph nodes, documented at the National Cancer Institute, a National Reference Center for Skin Tumors.

## Case presentation

**Figure 1:**
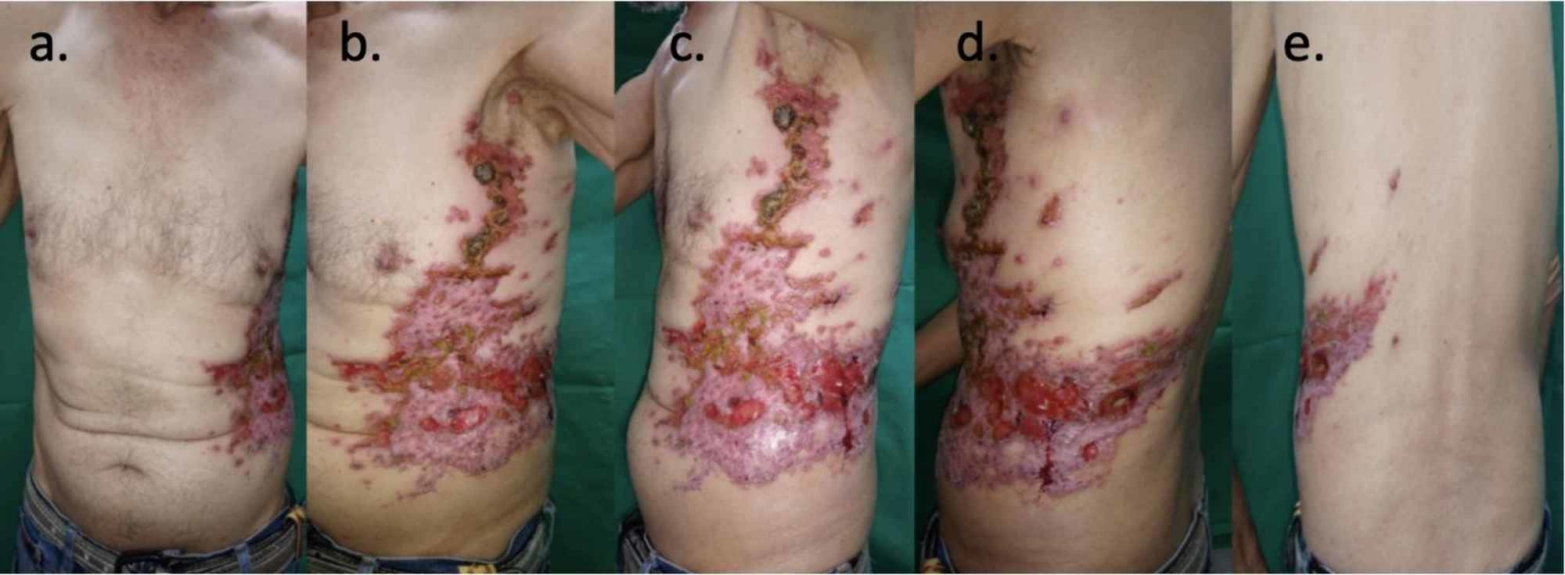
Clinical manifestation of zosteriform type metastatic eccrine porocarcinoma of the skin a - b: front view; c - d: lateral view; e - posterior view

A 64-year-old male, heavy smoker, with no other background of interest. He presented with the seven-year-old lesion on the skin of the lateral wall of the left hemithorax. It was initially described as a three-millimeter wart, which was empirically manipulated with its subsequent growth, progressively extending anteriorly and posteriorly in the cephalic and caudal direction, compromising from the armpit to the left flank. In the last two years, it was associated with ulceration, bleeding, peeling, weight loss, and functional limitation due to pain (Figure 1). Because it was initially diagnosed as herpes zoster by its characteristics and distribution, the patient received treatment with topical and oral acyclovir on multiple occasions during the last 12 months without improvement.

**Figure 2:**
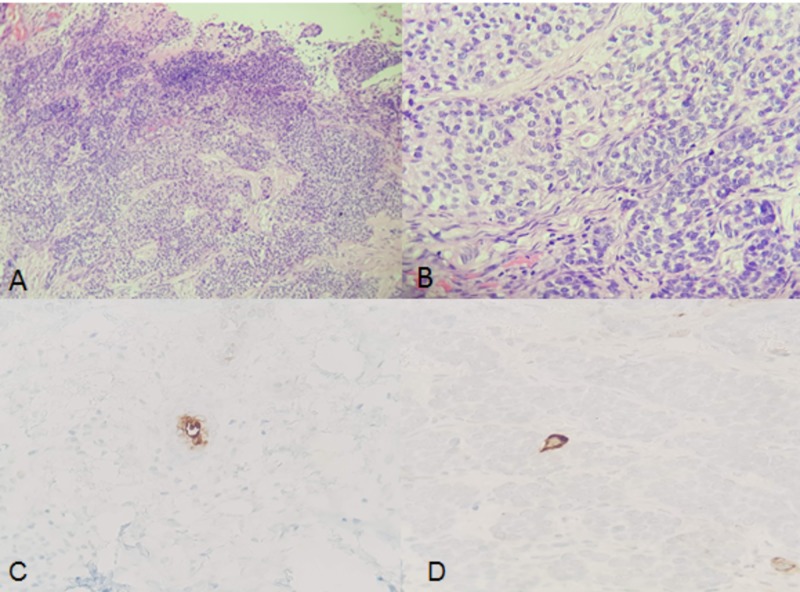
Porocarcinoma in the reported case A: Ulceration of the epidermis; anastomosed cords that connect to the epidermis (magnification 4x). B: Polygonal cells, with atypia and ductal differentiation (magnification 10x). C: Immunoreactivity with carcinoembryonic antigen (CEA). D: Immunoreactivity with epithelial membrane antigen (EMA).

A biopsy was performed with the following description in the pathology report: poorly differentiated squamous cell carcinoma of the trunk. The patient was admitted to the National Cancer Institute where a new biopsy was performed which observed: ulceration of the epidermis and malignant tumor lesion formed by cords of polygonal cells, atypical, with frequent anastomosed mitosis, with foci of ductal differentiation, which connect to the epidermis; the lesion occupies the entire thickness of the evaluated sample and has an infiltrative growth pattern (Figure 2, A-B). Immunohistochemical studies were performed with positive neoplastic cells for P63, epithelial membrane antigen (EMA), and carcinoembryonic antigen (CEA) at the duct level (Figure 2, C-D), and negative for CD117, S-100, CK7, chromogranin, and synaptophysin. With these findings, the diagnosis of ulcerated porocarcinoma was made, with a tumor thickness of 1.5 cm, a mitotic index of 10 mitosis/mm^2^, and an infiltrative and expansive growth pattern. 

**Figure 3:**
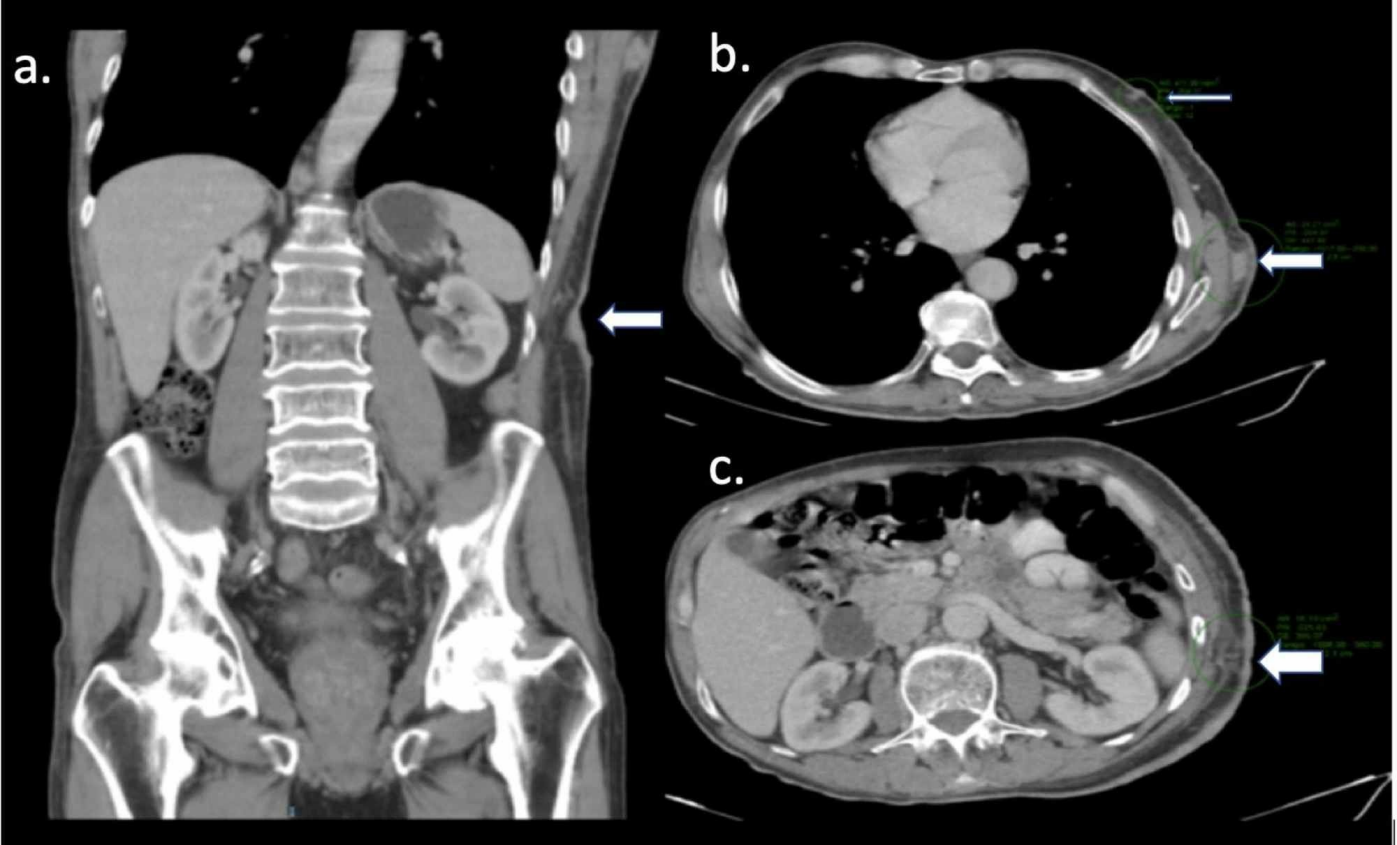
CT scan of the abdomen showing thickening of the skin and metastatic subdermal nodules (normal arrows on panels a - b) and ulcerated skin and soft tissue edema (thick arrow on panel c)

During a physical examination, a large skin involvement of zosteriform type, enlarged adenopathies suspicious of involvement in the armpit and ipsilateral inguinal region, was documented. Fine needle aspiration biopsy of suspicious axillary and inguinal adenopathies was performed with a positive report for malignancy. CT scans were performed with evidence of ipsilateral axillary and inguinal lymph node involvement, diffuse thickening of the skin and areas of tumor involvement of the subcutaneous tissue in the left hemiabdomen (Figure 3). No evidence of visceral metastatic involvement was found. The patient was treated with external radiotherapy, intensity-modulated radiation therapy (IMRT) technique, with a total dose of 54 Gy, sequentially with chemotherapy, based on a taxane and carboplatin regimen.

## Discussion

Eccrine porocarcinoma was first described by Pinkus and Mehregan in 1963 [3]. It is an uncommon cutaneous neoplasm that represents between 0.005% and 0.01% of all skin tumors [1]. Porocarcinoma arises from the intraepidermal portion of the duct of the eccrine sweat glands and can be either limited to the epidermis or spread to the dermis. According to gender, this type of carcinoma is distributed evenly (49% in men and 51% in women). Its average age of onset is the seventh and eighth decade of life (average age of 67 years) [2]. The most frequent clinical presentation is a nodule (71% of cases), associated with warty epidermal changes and/or ulceration. Most cases of porocarcinoma occur de novo and grow slowly or are stable for several years. Due to their slow growth, the lesions are usually asymptomatic, which causes a delay in diagnosis. It is estimated that 30% to 50% of porocarcinomas arise from an eccrine poroma. In these cases, tumors are characterized by rapid growth, ulceration, bleeding, or pain [4]. The average duration of the presentation is five years, and its most frequent location is head and neck (39%), and lower limbs (33%) [2]. Few reports in the literature describe cases of porocarcinoma in unusual places, such as vulva, soft tissue, and eyelid. Risk factors for developing porocarcinoma are not well known; several authors reported patients with immunodeficiency and others consider exposure to sunlight as a risk factor due to presentation in exposed areas (head, neck, hand, leg) [5, 6].

Porocarcinoma is considered a carcinoma with locally aggressive behavior and a high recurrence rate. In a review of 453 cases of porocarcinoma, it was found that 31% of the cases had concomitant metastases at the time of presentation and the most common organs are lymph nodes (57.7%), also evidenced in our case, followed by visceral metastases to lung (13%), liver (9.9%), brain (9.9%), skin (5.8%), bone (3.2%) and stomach (0.6%). It is noteworthy that cutaneous metastases are a rare presentation of porocarcinoma and, more unusual, the zosteriform type, as presented in our case. As far as we know, there is a case published in 2015 by Müller et al. describing an unusual presentation of porocarcinoma with zosteriform cutaneous metastases confirmed with a biopsy and associated nodal involvement; adjuvant treatment with cetuximab was offered which was discussed but rejected by the patient [7]. Our case is an unusual metastatic presentation of the eccrine porocarcinoma, with an epidermotropic zosteriform type growth pattern. This pattern has been reported sporadically in ovarian adenocarcinoma and other types of tumors, such as squamous cell carcinoma [7, 8]. Zosteriform dissemination has been attributed to direct lymphatic infiltration of cancer cells [7, 9].

In most cases, porocarcinoma is histologically diagnosed rather than clinically. From the histology point of view, it is a tumor that connects to the epidermis and is characterized by the proliferation of atypical polygonal cells arranged in anastomosed cords, nests or trabeculae with infiltrative or pushing edges. By definition, it has ductal differentiation and is positive for CEA and EMA. Adjacent eccrine poroma can be identified by up to 20%, suggesting a transformation of a benign lesion. Squamous differentiation of clear cells, sarcomatoid change, and melanin pigment can be identified. Histopathologically speaking, the main differential diagnoses are squamous cell carcinoma, hidradenocarcinoma, and extracutaneous metastatic carcinomas [10, 11].

Due to the rarity of the neoplasm, there are no established treatment algorithms. Despite this, surgical resection is considered to be the mainstay of treatment offering a cure rate of 70% to 80%, and a recurrence rate at the local and/or regional level of 20%, with a global survival at five years of 67% with lymph node involvement [12, 13]. It is known that porocarcinoma is an aggressive tumor, with an important nodal involvement at the time of diagnosis; due to this reason, the need to perform sentinel node biopsies in patients without clinically nodal involvement has been raised. Although there is no treatment guideline, it has been considered in patients with the presence of certain risk factors such as invasion (depth) greater than 7 mm, high mitotic rate, and the presence of lymphovascular invasion [4]. Surgery is the basis of the treatment in resectable patients; the use of Mohs micrographic surgery for surgical resection with safe oncological margins has been suggested due to high recurrence rates and local aggressive behavior [14]. Prophylactic lymph node dissection is controversial but seems to have no impact on disease-free survival [15, 16]. Treatment with radiotherapy and chemotherapy has been reserved for cases with a metastatic or recurrent disease as is our case [12, 16]. But it has also been recommended in high-risk patients (with tumors larger than 5 cm, positive surgical margins, poorly differentiated tumors with lymphovascular invasion), and in lymph node involvement with extranodal extension or involvement greater than four nodes [15].

The treatment of metastatic forms is not well described as being considered a chemoresistant carcinoma [17, 18]. Different therapeutic regimens have been proposed with variable results and based on 50% of cases in platinum, with global survivals of three to 35 months [12, 15]. Docetaxel, as a single agent, has been used in metastatic eccrine porocarcinoma with symptomatic and radiological response lasting for several months and with good tolerance [19, 20]. Taxane and carboplatin regimen concomitant with radiotherapy was used in our case.

## Conclusions

Porocarcinoma is a rare carcinoma, little known and madly aggressive, with a high recurrence rate and metastatic involvement. The mainstay of treatment is surgical resection in resectable patients and, for those with metastatic involvement, chemotherapy, and radiotherapy are the therapeutic options. It should be acknowledged that zosteriform metastasis is a presentation of the unusual porocarcinoma that requires a multidisciplinary approach and treatment. 
